# Framework for enhancing clinical practice guidelines through continuous patient engagement

**DOI:** 10.1111/hex.12467

**Published:** 2016-04-26

**Authors:** Melissa J. Armstrong, Juan‐David Rueda, Gary S. Gronseth, C. Daniel Mullins

**Affiliations:** ^1^ Department of NeurologyUniversity of Florida College of MedicineGainesvilleFLUSA; ^2^ Department of NeurologyUniversity of Maryland School of MedicineBaltimoreMDUSA; ^3^ Pharmaceutical Health Research DepartmentUniversity of Maryland School of PharmacyBaltimoreMDUSA; ^4^ Department of NeurologyUniversity of Kansas Medical CenterKansas CityKSUSA

**Keywords:** decision‐making, guidelines as topic, guideline adherence, patient participation, patient‐centred care

## Abstract

**Background:**

Patient engagement in clinical practice guideline (CPG) development is recommended by multiple institutions and instruments measuring guideline quality. Approaches to engaging patients, however, vary between oversight organizations, quality tools and guideline developers.

**Objective:**

We propose a ten‐step framework outlining steps and options for patient engagement in guideline development with the goal of highlighting steps for patient engagement and methods by which this can be achieved.

**Discussion:**

This framework provides a model for continuous patient engagement in CPGs by outlining ten steps of guideline development occurring at the levels of the developer/committee and the individual guideline project. At the developer level, patients can assist in topic nomination (step 1), topic prioritization (step 2) and guideline development group selection (step 3). Within specific guideline projects, patients’ opinions may be incorporated when framing the question (step 4), creating an analytic framework and research plan (step 5), conducting the systematic review and conclusion formation (step 6), development of recommendations (step 7) and dissemination and implementation (step 8). At the end of process, patients can again be engaged at the developer level by helping determine when guidelines need updating (step 9) and evaluating the developer's approach to patient engagement (step 10).

**Conclusions:**

Patient engagement at each CPG development step has different purposes, mechanisms, advantages and disadvantages, and implications for resource utilization. This framework can serve as a resource for guideline developers desiring to increase patient engagement and reference for researchers investigating engagement methodology at different steps of the CPG lifecycle.

## Background

International guideline standards describe patient engagement as a key element of high‐quality evidence‐based clinical practice guidelines (CPGs). The Appraisal of Guidelines for Research and Evaluation II (AGREE II) instrument for evaluating CPGs requires that guideline developers seek the views of the target population,^1^ the World Health Organization (WHO) recommends stakeholder involvement if ‘feasible and efficient’,^2^ and other bodies recommend inclusion of patients on guideline development groups (GDGs)^3^, ^4^, ^5^ (Table 1). Rationales for patient engagement in CPGs include recognizing patients as experts with important contributions, empowering consumers in well‐informed healthcare decisions and respecting the rights of citizens in healthcare policy; goals include the development of more patient‐centred and trustworthy guidelines that lead to improved implementation and quality of care.^6^

**Table 1:** Patient engagement requirements/recommendations from select groups describing standards for guideline development

Group	Recommended patient engagement approach
AGREE II^1^	‘5. The views and preferences of the target population (patients, public, etc.) have been sought’ (part of Domain 2. Stakeholder Involvement)
G‐I‐N International Standards^3^	‘A guideline development panel should include diverse and relevant stakeholders, such as health professionals, methodologists, experts on a topic and patients or other healthcare consumers’
IOM^5^	Standard 3.1: ‘The GDG should be multidisciplinary and balanced, comprising a variety of methodological experts and clinicians, and populations expected to be affected by the CPG.’ Standard 3.2: ‘Patient and public involvement should be facilitated by including (at least at the time of clinical question formulation and draft CPG review) a current or former patient and a patient advocate or patient/consumer organization representative in the GDG’. Standard 3.3: ‘Strategies to increase effective participation of patient and consumer representatives, including training in appraisal of evidence, should be adopted by GDGs'.Standard 7.1: ‘External reviewers should comprise a full spectrum of relevant stakeholders, including scientific and clinical experts, organizations (e.g. health care, specialty societies), agencies (e.g. federal government), patients and representatives of the public’. Standard 7.4: ‘A draft of the CPG at the external review stage or immediately following it (i.e. prior to the final draft) should be made available to the general public for comment. Reasonable notice of impending publication should be provided to interested public stakeholders’.
NICE^4^	All GDGs are expected to include at least two patient/caregiver/advocate members. Patient organizations can also register to provide stakeholder comments on drafts, nominate patient/caregiver members to the GDG and submit evidence.
WHO^2^	‘How should WHO ensure that appropriate values are integrated in recommendations? All WHO guideline groups should uniformly apply explicit, transparent and clearly described methods for integrating values. WHO should consider involving relevant stakeholders if this is feasible and efficient. WHO should develop a checklist for guidelines panels to help them to ensure that ethical considerations relevant to recommendations are addressed explicitly and transparently. How should users and consumers be involved in generating recommendations? Including consumers in groups that are making global recommendations presents major challenges with respect to the impossibility of including a representative spectrum of consumers from a variety of cultures and settings. Nonetheless, consideration should be given to including consumers in groups who are able to challenge assumptions that are made about the values used for making recommendations, rather than represent the values of consumers around the world. WHO should establish a network to facilitate involvement of users. Draft recommendations should be reviewed by consumers, who should be asked explicitly to consider the values that were used’

AGREE, Appraisal of Guidelines for Research and Evaluation; G‐I‐N, Guidelines International Network; IOM, Institute of Medicine; GDG, guideline development group; CPG, clinical practice guideline; NICE, National Institute for Clinical Excellence; WHO, World Health Organization.

While the tendency to include patients’ views is clear, guidance on ‘who’, ‘how’ and ‘when’ varies with no evidence guiding best practice. For the ‘who’, strategies include engaging patients, caregivers/family members, advocates, and/or consumers, with different reasons these approaches may be preferable. Various approaches also exist for recruiting representatives who will express not only their own views but also those of others. In this Viewpoint, however, we focus on a framework outlining possibilities for the ‘how’ and the ‘when’ of patient engagement in CPGs. We use the term ‘patient’ to refer to all of the lay stakeholders filling this role, consistent with other frameworks.^7^

In considering the ‘how’ of patient involvement, the Guidelines International Network (G‐I‐N) PUBLIC Toolkit describes strategies and outlines three potential approaches: (1) consultation, where patient preferences are collected through direct consultation or review of published literature, (2) participation, where patients participate in GDGs, and (3) communication, where information is provided to the public through lay‐targeted materials.^6^ As examples of soliciting views and preferences of target populations, AGREE II lists literature reviews of published preferences, formal consultations/interviews, participation on GDGs and external review.^1^ No research exists regarding best practices for the ‘how’ of obtaining patients’ views or whether single strategies are sufficient.

Additionally, there is variation in views regarding the ‘when’ of soliciting patient preferences. The Institute of Medicine (IOM) standards require that patients participate in GDGs, but qualify this by saying that GDG patient participation should be ‘at least at the time of clinical question formulation and draft CPG review’.^5^ AGREE II does not specify the times for solicitation of patient views.^1^ A review found that patients are most commonly engaged in CPG development during knowledge synthesis, recommendation development and draft revision,^8^ but patients may also be engaged in CPG development decisions (e.g. scope, GDG participants)^8^ or creation of lay‐targeted guideline materials.^4^, ^8^, ^9^ Most discussions of patient engagement relate to involvement at the level of the individual guideline rather than development steps occurring at developer or committee levels.

Given that optimal approaches for the methods and timing of patient engagement in CPG development are unknown, having a framework outlining guideline development and potential patient contributions at each step is critical. We thus propose a framework for patient engagement in guideline development (Table 2) that can facilitate discussion of continuous patient engagement (i.e., at developer, committee and individual guideline levels) and allow guideline developers and researchers to identify steps at which patients are engaged and methods by which this can be achieved. This ten‐step framework resulted from collaboration between the developer of the framework for continuous patient engagement in comparative effectiveness research^10^ and a guideline methodologist researching patient engagement in CPGs. Iterative revisions resulted from suggestions from others with guideline expertise. The framework includes common guideline development steps and optional (non‐exhaustive) methods for patient engagement at each step based on a review of published literature and pragmatic experience.

**Table 2:** Steps for continuous patient engagement in clinical practice guideline development

Step in guideline process	Purpose of patient engagement	Methods of patient engagement
1. Nominating guideline topics	Identify topics that are important to patients, caregivers, and the community Propose topics to be investigated	Directly solicit topic nominations from public Solicit topic nominations from patient advocacy groups Review priorities published by patient advocacy groups Review research on patients’ priorities and needs
2. Prioritizing guideline topic nominations	Solicit feedback on relevance and priority of topics Discuss the urgency of addressing topics	Survey patient groups Review research on patients’ priorities and needs Engage patients on guideline committees determining priorities^a^
3. Selecting guideline development group members	Help ensure that the GDG composition is both representative and trustworthy Assess conflicts of interest of panel members from patient perspective	Review proposed panel members’ conflicts of interest Approve proposed panel with ability to suggest changes Directly engage patients, caregivers and advocates on selection of guideline development group members^a^
4. Framing the question (including selection of comparators and outcomes)	Ascertain questions’ relevance and usefulness Assess ‘real‐world’ applicability Identify outcomes of relevance to patients, caregivers, and the community Incorporate other aspects of treatment	Perform focus groups on identified guideline topics Review existing research on patients’ priorities and opinions Solicit public comment on guideline topics prior to formalization of questions Ask stakeholders to suggest materials about patient preferences that are not formally published (‘grey literature’) Survey patients to rate importance of proposed outcomes Post draft research plan for public comment/review Directly engage patients, caregivers and advocates on GDGs^a^
5. Creating analytic framework and research plan	Help refine or expand scope of topic Identify potential harms associated with the questions posed Provide a ‘reality check’ Verify logic of analytic framework Supplement with additional factors not documented in the literature Discuss proxies for a specific concepts (e.g. whether test scores and school performance are interchangeable) Suggest additional search terms Inquire about potential confounding factors Identify particular populations of interest and/or important multimorbidity to consider in search	Review existing research on patients’ priorities and opinions Survey patients to rate importance of elements of proposed framework Post draft research plan for public comment/review Perform focus groups Directly engage patients, caregivers and advocates on GDGs^a^
6. Developing systematic review and forming conclusions	Assist with critical appraisal of studies and evidence synthesis Assess believability of results Suggest alternative interpretations of evidence	Solicit feedback on draft evidence review from guideline development group lay participants even if they did not participate in analysis of evidence Post draft evidence review for public comment Directly engage patients, caregivers, and advocates on GDGs^a^
7. Developing recommendations	Assist in translating evidence‐based conclusions into meaningful, clear, and respectful recommendations Assist in ensuring that recommendations foster partnership between physicians, patients and families Describe variability in patient preferences Help make recommendations easy to understand Provide input when there are gaps in the evidence Indicate which recommendations are counterintuitive (e.g. so that additional explanation can be provided)	Review existing research on patients’ preferences Post draft recommendation statements for public comment Perform focus groups Directly engage patients, caregivers and advocates on GDGs^a^
8. Disseminating and implementing recommendations	Endorse guidelines from patient perspective (either individually or in representation of patient groups) Assist in developing patient‐ and family‐level summaries of systematic review findings and guideline recommendations Assist in developing patient decision aids Identify barriers to implementation and possible solutions Facilitate engagement of other patients in dissemination Improve legitimacy and trustworthiness of guideline process such that recommendations are more likely to be implemented	Consult patients, caregivers, and advocacy groups regarding barriers to dissemination and implementation and identifying solutions Directly engage patients, caregivers and advocates in development of lay summaries and patient decision aides^a^ Engage individuals and advocacy groups in dissemination strategies
9. Updating	Identify when public or stakeholder views have changed such that a guideline requires update or reaffirmation	Solicit patient views regarding when guidelines need updating (e.g. on websites) Include patients in formal review of evidence regarding guideline currency^a^
10. Evaluating methods and impact of engagement	Identify if patients were engaged in a meaningful way Suggest options for improvement in future engagement strategies	Provide feedback regarding engagement experience Discuss feedback from participating patients (e.g. verbal, survey)

a May require additional training. GDG, guideline development group.

## Framework

There are opportunities for patient engagement throughout the guideline process. In this proposed ten‐step framework (Table 2), we include steps typically performed at the developer or committee level (Steps 1–3, 9, 10) and those which relate to specific guidelines (Step 4–8).

As a first step (Step 1), patients can identify important guideline topics. Determining priority topics is one method for patient engagement identified by AGREE II.^1^ Many guideline developers have websites allowing the public to submit topic nominations. How often passive routes allowing topic nomination engage patients, however, is unclear. Active solicitation of guideline topics through community meetings, focus groups or contacting advocacy organizations may be more likely to successfully engage patients. Given limited resources, however, guideline developers are unable to accept every nomination. Engaging patients in prioritizing nominated topics and selecting topics to proceed to guideline development (Step 2) can ensure that guideline developers are addressing target population needs. While engagement of this type is not widely described, the National Institute for Clinical Excellence (NICE) consults the public on whether particular technologies should be reviewed prior to initiating guideline development.

Once topics are accepted for development, patients can be involved in key process decisions including proposing and vetting GDG members (Step 3), framing the question (Step 4) and developing the research plan (Step 5). It is likely that if patient engagement impacts ultimate implementation success, engaging patients at early stages will be particularly important. The challenge of conflicts of interest (COIs) in guideline development is subject to on‐going debates with implications for guideline bias and end‐user trust. Assessing and managing COIs are thus critical parts of guideline development.^1^, ^3^, ^5^ Patients engaged in assessing COIs could provide a unique and novel voice in this process. Patients could also contribute meaningfully to developing guideline questions (Step 4). While limited research exists regarding the impact of patient engagement on CPGs, a descriptive study found that patient engagement resulted in the introduction of a new guideline subtopic.^9^ As part of question development, the Grading of Recommendations Assessment, Development and Evaluation (GRADE) working group suggests that GDGs rate the significance of patient‐important outcomes,^11^ a process that is clearly more meaningful with patients involved. At the research plan step (Step 5), patients’ input can help determine key elements to consider in the analytic framework (e.g. harms), suggest whether there are proxies for framework concepts and identify special populations of interest (e.g. those with comorbidities). This can be accomplished through reviewing literature regarding patient preferences and direct engagement through patient GDG participation, but it is common for guideline developers (e.g. United States Preventive Services Task Force [USPSTF], NICE) to also seek patient involvement at this stage through posting of research plans for public comment.

The degree to which patients should be engaged in the completion of the systematic review (Step 6) is debated, with some guideline developers describing active patient participation^8^ but others describing this as a barrier to successful patient engagement.^4^, ^8^, ^12^ NICE identifies the ability to understand scientific articles (with training) as part of the minimum skill set for patient and caregiver GDG participants^4^ and organizations like Consumers United for Evidence‐based Healthcare (CUE) can provide consumers with training on critical appraisal and evidence‐based medicine. However, requiring patient GDG members to participate in formally grading systematic review evidence may limit individuals’ interest in participation (based on time requirements, skill set) and result in underrepresentation of less educated populations whose views and opinions are important to solicit. Approaches to this include having GDG patient representatives who do not participate in the systematic review but contribute at other steps or providing training for GDG members. Posting of draft evidence summaries and conclusions for public comment feedback, particularly for face validity and meaningfulness, is another route for engaging patients at this step of the guideline process and is an approach already utilized by some developers (e.g. USPSTF).

There is near‐universal agreement that guideline developers need to engage patients in recommendation development (Step 7). The patient role in recommendation development is broad (Table 2), with contributions particularly relating to providing insight into how patient preferences inform recommendations and ensuring that recommendations facilitate patient‐centred care. Given patients’ key role in recommendation development, guideline developers may use multiple strategies for patient engagement particularly at this step, including direct engagement through GDGs and obtaining diverse opinions through posting of draft recommendations for public comment.

A key guideline step in which patients are commonly engaged is that of dissemination and implementation (Step 8). Patient engagement throughout all steps hopefully results in guidelines with terminology and language that patients use and value such that less rephrasing is required for implementation and clinician use. Patients clearly play an important role in developing lay language guideline versions and other public products,^4^, ^8^, ^9^ but their engagement at this step should not be limited to this one contribution. Some organizations accept patient endorsement of guidelines to demonstrate patient support for recommendations. Positive endorsement may rely on patient engagement at prior steps: articles describe guideline endorsement after successful patient engagement^9^ and also failure of endorsement when an engaged patient participant felt that his contribution was not valued.^12^ Engaging patients and patient advocacy groups in dissemination could also be a key avenue for improving guideline awareness and implementation.

Quality CPGs have plans for currency reviews and updates^1^, ^3^, ^5^ (Step 9). As with topic nomination, patients can assist in this step by helping determine whether and when guidelines require update, particularly if there is a social or public reason for update apart from new evidence. Select guideline developers have processes for this in place, including the USPSTF, which allows the public to submit requests for topic reconsideration, and NICE, which invites review consultations when guideline update decisions are being made.

Finally, guideline developers should have mechanisms in place to evaluate the success of their patient engagement strategies (Step 10). In this setting, patients can help determine whether their contributions were meaningful and strategies for improved future participation.

## Barriers

While there are opportunities for patient involvement at each guideline development stage, barriers exist. A commonly cited barrier is the ability of patients to understand medical terminology and participate meaningfully in assessing research quality.^3^, ^4^, ^8^, ^12^, ^13^ Other barriers include recruitment difficulties,^8^ inadequate training and support,^4^, ^12^, ^13^ failure to engage patients in determining guideline scope before including them on GDGs,^4^ conduct of GDG meetings (including resistance to patient involvement),^4^, ^8^, ^13^ the fact that patients on GDGs may not know or represent other patients’ views,^4^, ^8^ the commitment required (work, time),^8^ discrepancies between the views of patients and physicians,^8^ uncertainty of how to incorporate patient experiences into evidence‐based guidelines^12^ and the resources needed to engage patients well.^8^, ^12^ When patients are actively engaged in the guideline process (as opposed to relying on literature reviews, surveys and public comment), training is key to meaningful involvement.^3^, ^4^, ^6^, ^8^, ^14^

## Conclusions

Opportunities exist for patient engagement throughout guideline development, both in steps occurring at the developer level and steps that are guideline project specific. Patient engagement at each step has different purposes, mechanisms, advantages and disadvantages, and implications for resource utilization. Guideline developers need to thoughtfully consider patient engagement at each step; specific goals may dictate types of engagement utilized. Engaging patients through multiple mechanisms – for example, GDG participation and public comment – has advantages and may be a particularly important strategy for addressing barriers such as GDG members representing only select views. Using multiple engagement strategies (e.g. literature review, direct representation on GDGs and public comment) allows for increased representation of patient views but also allows developers to prioritize limited resources for certain steps and respect patients’ time and work capacity by actively engaging them at targeted steps while using alternate strategies (e.g. systematic literature reviews of patient preferences, public comment) for other steps. For certain strategies, additional training may be needed for both patients and the panels that are engaging them. Resource availability, time sensitivity and disease‐specific considerations (e.g. cognitive impairment) may influence when and how guideline developers engage patients. It remains uncertain whether continuous patient engagement at each of the ten steps has superior outcomes vs. engagement only at select steps. It is hoped that engaging patients will enhance the validity and usefulness of published guidelines. The result will be CPGs that are more meaningful to patients, thus improving clinical practice, decision making and patient‐relevant outcomes.

## Conflicts of interest

None.

## Source of funding

MJA is supported by an Agency for Healthcare Research and Quality (AHRQ) K08 career development award (K08HS24159‐01) for research on patient engagement in clinical practice guidelines, through which this manuscript was developed.
